# Correlation between the color stability and contact profilometry results of different CAD/CAM ceramic materials after staining with black shammah and DZRT smokeless tobacco

**DOI:** 10.18332/tid/215947

**Published:** 2026-04-01

**Authors:** Mohammed Hussain AlWadei

**Affiliations:** 1 Department of Restorative Dental Science, College of Dentistry, King Khalid University, Abha, Saudi Arabia

**Keywords:** smokeless tobacco, CAD/CAM ceramics, color stability, black shammah, DZRT snuff

## Abstract

**INTRODUCTION:**

This *in vitro* study investigated the effects of two types of smokeless tobacco (ST), black shammah and DZRT snuff, on the mean color stability (ΔE**) and contact profilometry results (surface roughness [R_a_]) of five Computer-Aided Design and Computer-Aided Manufacturing (CAD/CAM) ceramic materials: feldspathic ceramics (VITABLOCS TriLuxe Forte and Mark II), multilayer zirconia (Ceramill Zolid PS), zirconia-reinforced lithium silicate (VITA Suprinity), and lithium disilicate glass ceramic (IPS e.max CAD).

**METHODS:**

A total of 100 specimens of VITABLOCS Mark II, Ceramill Zolid Multilayer PS, VITA Triluxe Forte, IPS e.max CAD, and VITA Suprinity were divided into two ST exposure subgroups, the black shammah and DZRT snuff groups. Samples underwent daily tobacco staining, thermocycling (5–55°C), and brushing simulation (10000 cycles). Color parameters (L*, a*, and b*) were measured by using a spectrophotometer (VITA Easyshade^®^ Compact) at baseline and at one, two, and four weeks. ΔE** was calculated by employing the CIEDE2000 formula. Assessments with the VITA classical shade guide were employed to record shifts in the lightness/darkness of samples. R_a_ (μm) was assessed via contact profilometry at the end of the study. Statistical analyses included the Kruskal–Wallis, Mann–Whitney U, and chi-squared tests, with significance set at p<0.05.

**RESULTS:**

After four weeks of immersion in DZRT snuff, IPS e.max CAD showed the highest ΔE** (6.718) among the tested materials, whereas Ceramill Zolid PS and VITABLOCS Mark II exhibited the lowest ΔE** values (0.614–1.075) across ST types and durations. Significant ΔE** differences were observed among ceramics (p<0.05), staining solution types (p<0.001 for IPS e.max CAD at four weeks), and immersion times (p<0.001). Assessments with the VITA classical shade guide showed that shades darkened significantly (p<0.05) after 2–4 weeks (p<0.05), with the prevalence of the A2 shade decreasing from 62% to 42% after exposure to black shammah and from 58% to 50% after immersion in DZRT snuff. Among materials, VITABLOCS Mark II had the lowest Ra (1.61–1.65 μm) and IPS e.max CAD had the highest (2.89–2.92 μm).

**CONCLUSIONS:**

Prolonged exposure to ST induced clinically significant color changes in IPS e.max CAD. Meanwhile, Ceramill Zolid PS and VITABLOCS Mark II demonstrated superior color stability over other materials. Material selection for ST users should prioritize zirconia or feldspathic ceramics to minimize aesthetic compromise.

## INTRODUCTION

Aesthetic dentistry has advanced remarkably, particularly with the development of ceramic prostheses, like VITABLOCS TriLuxe Forte, a feldspathic ceramic with enhanced strength and layered shading for natural aesthetics, and VITA Suprinity, a zirconia-reinforced lithium silicate ceramic known for its durability and appearance^1^. Lithium disilicate glass ceramics (IPS e.max CAD), feature a 70% crystalline matrix and are valued for their biocompatibility and resistance to wear^2^. Ceramill Zolid PS zirconia has a unique microstructure, grain size, and grain distribution, resulting in a marked effect on its clinical performance and survivability^3^.

These ceramics are widely used in crowns, bridges, veneers, inlays, onlays, overlays, and vonlays for restorative dentistry to achieve optimal aesthetics^4^.

The global burden of smokeless tobacco (ST) consumption represents a crucial public health challenge. According to WHO estimates, ST is used by >1.3 billion users worldwide despite being a preventable cause of oral cancers and precancerous conditions^5,6^. These products typically consist of locally grown tobacco varieties, like *Nicotiana rustica* and *Nicotiana glauca*, processed with additives, including ash, plant oils, calcium hydroxide, and spices^7^.

This heterogeneous mixture is typically held in the oral vestibule for periods ranging from approximately 7 to 10 min per use. In Saudi Arabia, traditional ST products, like black shammah, remain common, while new commercial options, such as DZRT snuff, are becoming increasingly popular, especially in southern regions, such as Jazan, Najran, and Asir, with concerning use among adolescents aged 13–17 years^8^. Alkhatib et al.^9^ found that ST consumption leads to characteristic brown tooth staining on anterior teeth at contact sites.

A critical factor in determining the success of dental restorations is their ability to maintain color stability over time^10^. Researchers rely on advanced color measurement tools, such as spectrophotometers, which capture precise color data and convert them into numerical values for analysis, to quantify color changes objectively^11,12^. The most widely used system for color assessment is the CIELab (L*, a*, b*) color space established by the Commission Internationale de l’Eclairage; this system defines the mean color change (ΔE^**^) as the standard for color-matching perception^13^. The CIELab color space consists of lightness (L*), red–green (a*), and yellow–blue (b*). In clinical settings, a ΔE value exceeding 4.2 is typically deemed unacceptable because it indicates a visually perceptible and undesirable color change^14^. New formulas, like CIEDE2000, improve accuracy by adjusting for lightness, chroma, and hue. Recent studies have indicated that CIEDE2000 provides good indicators of the human perceptibility and acceptability of color differences in tooth shades^15^.

Various studies have examined the effects of culture-specific practices, such as the use of khat, ST, and snuff and consumption of coffee, on the ΔE^**^ of CAD/CAM ceramics^13,14^. Among ceramics, zirconia-reinforced lithium silicate (VITA Suprinity) has the lowest color stability under coffee and tea exposure, whereas lithium disilicate (IPS e.max CAD) shows superior resistance^16^. Monolithic zirconia (Ceramill Zolid PS) demonstrates minimal color change (ΔE^**^ =1.8–2.3) under ST exposure, with its ΔE^**^ remaining below the unacceptable level (ΔE^**^ >4.2). By contrast, feldspathic ceramic (VITA TriLuxe Forte) and VITA Suprinity have high ΔE^**^ values of 4.07–4.87^17^. IPS e.max CAD shows unacceptable discoloration (ΔE^**^ >3.5) from ST and yerba mate (ΔE^**^ =7.6) and slight color change under exposure to snuff^18^.

The longevity of prosthetic materials depends on their surface roughness^19^. Excessive surface roughness (R_a_ >0.2 μm) can cause discoloration and biofilm accumulation^14^. Research on the effects of ST on dental ceramics has reported marked material differences. Al Moaleem et al.^17^ found that two weeks of exposure to black and white ST increased the R_a_ of VITA Triluxe Forte to 0.41 and 0.48 μm, respectively. By contrast, Ceramill Zolid PS showed good resistance, with R_a_ values of 0.30 (after one week of exposure to black ST) and 0.32 μm (two weeks of exposure to white ST)^17^. Alqahtani et al.^20^ noted that VITA Suprinity and VITABLOCS Mark II had lower roughness than other ceramics after exposure to ST and thermocycling. Moreover, glazed surfaces exhibit better color stability and lower roughness changes than unglazed or poorly polished ones^19^.

VITA classical shade guide was developed in the 1950s, remains a widely used tool for visual shade matching in dental clinics across Saudi Arabia, particularly in settings where advanced devices, such as spectrophotometers, are unavailable. This shade guide organizes tooth shades into four distinct groups: A1–A4 (reddish-brownish), B1–B4 (reddish-yellowish), C1–C4 (grayish), and D2–D4 (reddish-gray)^21^. Practitioners must maintain standardized conditions, including light source, illumination angle, and distance between the light and tooth surface, to ensure accurate and consistent shade selection^22^. Research has demonstrated that CAD/CAM ceramics exhibit color changes when exposed to staining solutions. Specifically, shades originally matching A1 and B1 (light tones) on the VITA classical guide may shift toward dark shades, like D2 and B2, after immersion in staining agents^17,20^.

To our knowledge, no single study has comprehensively assessed the effects of two types of ST on the mean color stability (ΔE^**^), contact profilometry (R_a_), and VITA classical shade guide changes of CAD/CAM ceramics. In this *in vitro* study, we evaluated the effects of two ST products, black shammah and DZRT snuff, on the mean color stability (ΔE^**^), and contact profilometry results (R_a_), of feldspathic ceramics (VITABLOCS TriLuxe Forte and VITABLOCS Mark II), multilayer zirconia (Ceramill Zolid PS), zirconia-reinforced lithium silicate (VITA Suprinity), and lithium disilicate glass ceramic (IPS e.max CAD) over exposure periods of one, two, and four weeks. Our null hypothesis states that the ΔE^**^ of the five CAD/CAM ceramic materials will not significantly differ after exposure to black shammah and DZRT snuff for one, two, or four weeks and is not significantly affected by staining agent type or immersion duration. Additionally, staining agents have no effects on the VITA classical shade of the tested CAD/CAM ceramics over one, two, or four weeks of treatment.

## METHODS

### Study design and sample size calculation

This *in vitro* study assessed the effects of two types of ST, black shammah and DZRT snuff, on the mean color stability (ΔE^**^) and contact profilometry results (R_a_) of five types of CAD/CAM ceramic materials over one, two, and four weeks of exposure. The sample size was determined a priori using G*Power software (version 3.1; University of Dusseldorf). The primary outcome was the color change (ΔE) after four weeks of ST immersion, based on data from previous studies investigating ST effects on dental ceramics^17,18,23^. An anticipated medium effect size (f=0.35) was estimated from the reported ΔE^**^ differences among ceramic groups exposed to smokeless tobacco. With an alpha level of 0.05, a power (1–β) of 0.80, and five independent material groups, the analysis indicated a minimum requirement of 10 specimens per group. To account for subgroup stratification by ST type (black shammah and DZRT snuff) and to ensure robustness for ΔE^**^ and contact profilometry measurements (R_a_)^24^, a total of 100 specimens (20 per ceramic material, 10 per ST subgroup) were prepared.

### Sample preparation

The CAD/CAM ceramic restorative materials VITABLOCS Mark II, Ceramill Zolid Multilayer PS, VITA Triluxe Forte, IPS e.max CAD, and VITA Suprinity were used to prepare the study samples. A total of 100 circular specimens were prepared, with 20 specimens per material.

All specimens were milled to standardized circular dimensions (10.0 ± 0.1 mm diameter × 2.0 ± 0.1 mm thickness) by using a Ceramill Motion CAD/ CAM system (Amann Girrbach GmbH, Pforzheim, Germany) by a single operator. Surface finishing was performed with silicon carbide abrasive paper (300–800 grit sequence under water cooling). Before undergoing glazing and sintering, the specimens were ultrasonically cleaned in distilled water for 10 min, degreased in isopropanol for 5 min, and dried with oil-free compressed air.

### Glazing, sintering, and grouping of samples

Material-specific postprocessing was rigorously applied in accordance with the manufacturers’ instructions. VITAblocs Mark II and VITA Triluxe Forte were applied with a glaze (VITA Glaze LT; VITA Zahnfabrik, Bad Säckingen, Germany) and heated from 600°C to 900°C over 9 min, with holding at 900°C for 1 min. Ceramill Zolid multilayer PS specimens were initially sintered at 900°C for 30 min, then sintered at 1450°C for 120 min, and finally cooled down to 200°C. They were glazed with UniGlaze at an initial temperature of 440°C, with the temperature being increased to 815°C at the rate of 45°C per min and held for 1 min, then cooled for 3 min. IPS e.max CAD specimens were crystallized at 840°C for 30 min in an electric furnace (Programator P300, Ivoclar Vivadent, Liechtenstein). VITA Suprinity specimens were glazed with VITA AKZENT Plus at temperatures that started at 380°C for 4 min, then raised to 840°C over 8 min, and held for 8 min for crystallization and glazing.

The specimens were randomly further categorized on the basis of the type of ST exposure (black shammah and DZRT snuff), resulting in two subgroups of 10 specimens per material. The VITABLOCS Mark II, Ceramill Zolid multilayer PS (zirconia), VITA Triluxe Forte, IPS e.max CAD, and VITA Suprinity specimens exposed to black shammah and DZRT snuff were denoted as A, B, C, D, and E, respectively.

### Mean color evaluations

Color measurements were performed by using a VITA Easyshade^®^ Compact spectrophotometer (Version V; VITA Zahnfabrik, Bad Säckingen, Germany) with a 4 mm diameter tip. The device was calibrated after each measurement in accordance with the manufacturer’s protocol. Prior to staining, ceramic samples were ultrasonically cleaned in distilled water for 10 min and dried with compressed air.

Measurements were recorded by using the CIEDE2000 system at baseline (prestaining) and after one, two, and four weeks of exposure to black shammah and DZRT snuff and expressed as ΔE^**^. The probe tip was positioned perpendicularly (90°) to specimens under controlled lighting to minimize interference. L*, a*, and b* coordinates were measured three times for each sample at consistent daily times, with averages used for analysis^25^.

ΔE^**^ values were calculated by using the CIEDE2000 formula^15,24^, which accounts for luminosity (ΔL'), chroma (ΔC'), hue (ΔH'), and chroma–hue interactions (R_T_). Parametric correction factors (K_L_, K_C_, and K_H_) were set to 1 because of standardized lighting. The weighting factors SL, SC, and SH and correction coefficients KL, KC, and KH were applied to adjust for variations in L*, a*, and b* coordinates. The CIEDE2000 formula is:


CIEDE 2000=((ΔL′)2KLSL)+((ΔC′)2KCSC)+((ΔH′)KHSH)+RT((ΔC′)KCSC)+((ΔH′)KHSH)

Each color parameter was measured three times for each sample at similar daily times before staining with black shammah and DZRT snuff, and the average was recorded to characterize different color parameters^25^.

### VITA classical color parameter measurements

A blinded trained operator performed initial color assessments by using the VITAPan classical shade guide (VITA Zahnfabrik H. Rauter GmbH & Co. KG, Bad Säckingen, Germany) immediately after laboratory fabrication, establishing baseline (‘before’) measurements. Prior to evaluation, all specimens were gently rinsed with distilled water, then air-dried. Color analysis was conducted in strict compliance with ISO/TR 28642:2016 standards and VITAPan shade guide protocols by using a VITA Easyshade Compact spectrophotometer (version V) against a gray background to simulate intraoral lighting conditions. The specimens were re-evaluated for color stability following one, two, and four weeks of exposure to black Shammah and DZRT snuff. Any deviations from the original shade were classified as no change, darkening, or lightening^20^.

### Sample brushing and thermocycling

Brushing simulation was performed by using an electric toothbrushing simulation unit (model ZM-3.8, SD Mechatronik, Feldkirchen-Westerham, Germany), an Oral-B PRO 1000 brush head (Oral-B, Leicester, the UK), and a Colgate toothpaste slurry solution (250 g in 1 L of distilled water). The simulation was performed under a vertical load of 2.45 N (approx. 250 g), with the brush aligned and fixed by using a prefabricated stainless-steel holder at a rate of 180 strokes per min. A total of 10000 cycles of brushing simulation were performed at 5°C and 55°C, with a dwell time of 30 s in each bath; this simulation is equivalent to one year of clinical toothbrushing^26^. The brush head and toothpaste slurry were changed after 5000 cycles. The brush motion direction and pressure were standardized by the holder. Following each removal from staining ST media, circular samples were rinsed by dipping 10 times in distilled water, wiped dry with tissue paper, and allowed to air-dry completely before subsequent procedures.

### Immersion and staining of circular specimens

All glazed and sintered specimens were exposed to black shammah and DZRT snuff staining agents prepared in accordance with established protocols^17,18^. Each staining agent was mixed with distilled water to achieve a paste-like consistency. The black shammah paste was applied directly onto the circular specimens under a constant load for 10 min to simulate intra-oral pressure and typical exposure duration. The circular specimens for DZRT snuff, which users typically retain within a pouch-like material in the buccal vestibule where it interacts with saliva, were immersed directly between the DZRT snuff material and its simulated pouch liner. Similar constant load with similar pressure times to mimic existing intra-oral pressure during actual usage were applied^17,18^.

All specimens in both staining agents were also covered in artificial saliva throughout the experiment to simulate salivary interaction^18^. Both staining agents were replenished twice daily over exposure periods of one, two, and four weeks to maintain activity and reflect common consumption patterns. The above intervals were selected to approximate 12, 24, and 48 months of clinical usage^26^.

### Contact profilometry

The surface topography (R_a_) of randomly selected CAD/CAM specimens (n=3 per ceramic/staining group) was measured at the end of the study after color parameter (ΔE** and VITA Classical shade guide) measurement, staining, brushing, and thermocycling by using a profilometer (Contour GT-K1, Bruker Nano GmbH, Berlin, Germany). Triplicate profilometric readings were taken at the center of each circular sample under the following standardized conditions: a scanning speed of 0.25 mm/s and cutoff length of 0.80 mm^24^. The mean of these three measurements was calculated and recorded as the final R_a_ value (μm).

Supplementary file Table 1 shows the characteristics of the different materials and devices used in this in vitro study. Supplementary file Figure 1 displays the ceramic and ST employed in the present work.

### Statistical analysis

The mean ΔL, Δa, Δb, and ΔE** values at baseline and after one, two, and four weeks of exposure were calculated for quantitative variables. Frequencies and percentages were used for qualitative variables. SPSS version 26.0 (SPSS Inc., Chicago IL, the USA) was used to input and analyze the data. The Shapiro–Wilk test indicated that all quantitative variables were not normally distributed (p<0.05). Therefore, the medians and interquartile ranges (quartile 1 and 3) are presented. The Kruskal–Wallis test was used to compare ΔE** values among different ceramic types and different durations of exposure to black shammah. The Mann–Whitney test was used to compare ΔE** values between ST types.

Moreover, *post hoc* test with correction was used for pairwise comparisons between every two different ceramic types and different times after immersion in ST. The chi-squared test was used to compare the changes in VITA classical shades after one, two, and four weeks of immersion in ST with their baseline values. A p<0.05 was considered as a cutoff point for statistical significance.

## RESULTS

Table 1 gives the median (IQR) values of ΔL, Δa, Δb and ΔE** of ceramic types following exposure to black shammah and DZRT. After one week of exposure, the VITA Suprinity ceramic showed the highest ΔE** values (1.396 under black shammah and 1.395 under DZRT snuff) among the tested materials, followed by the VITA TriLuxe ceramic (1.218 under black shammah and 1.343 under DZRT snuff). After two weeks of exposure, IPS e.max CAD exhibited the highest ΔE** values (2.486 under black shammah and 2.904 under DZRT snuff) among the ceramics, followed by VITA TriLuxe (1.852 under black shammah and 2.241 under DZRT snuff). Similarly, after four weeks, IPS e.max CAD exhibited the largest ΔE** values (2.975 under black shammah and 6.718 under DZRT) among the materials, followed by VITA TriLuxe (1.192 under black shammah and 1.269 under DZRT snuff).

**Table 1:** Median values of ΔL, Δa, Δb, and ΔE** for five CAD/CAM ceramic types after different time intervals exposure and types of smokeless tobacco

*Time*	*Ceramic type*	*Smokeless tobacco*							
		*Black shammah*				*DZRT snuff*			
		*ΔL*	*Δa*	*Δb*	ΔE**	*ΔL*	*Δa*	*Δb*	ΔE**
**After one week**	VITABLOCS Mark II	-0.710	0.020	-1.630	0.971	-1.010	-1.750	-2.080	1.486
	Zirconia	-0.060	-0.570	-0.890	1.036	-0.740	-1.500	-1.670	1.189
	VITA TriLuxe	-0.290	-1.280	-1.970	1.218	0.980	-2.130	-1.180	1.343
	IPS e.max CAD	-1.330	-1.270	-1.180	1.134	-0.640	-2.100	-0.130	1.164
	VITA Suprinity	-0.870	-1.590	-1.980	1.396	-0.760	-2.010	-1.400	1.395
**After two weeks**	VITABLOCS Mark II	0.020	-0.820	-0.680	0.648	0.840	-2.800	-2.820	2.037
	Zirconia	0.370	-1.430	-0.320	1.049	1.090	-2.640	-2.750	2.109
	VITA TriLuxe	0.750	-3.350	-1.230	1.852	2.080	-2.600	-2.940	2.241
	IPS e.max CAD	-2.750	-1.750	3.350	2.486	-2.000	-2.610	4.600	2.904
	VITA Suprinity	-0.050	-2.070	-0.170	1.080	0.890	-2.830	-3.090	2.161
**After four weeks**	VITABLOCS Mark II	-0.260	-0.800	-0.400	0.623	-0.050	-1.130	0.510	0.971
	Zirconia	0.190	-1.550	-0.240	1.075	0.660	-0.140	0.800	0.614
	VITA TriLuxe	-0.320	-2.180	-0.360	1.192	2.050	-0.400	1.370	1.269
	IPS e.max CAD	-3.040	-1.620	4.810	2.975	-3.320	0.650	13.000	6.718
	VITA Suprinity	0.220	-2.340	0.190	1.207	0.360	-0.280	1.660	0.925

**Figure 1:**
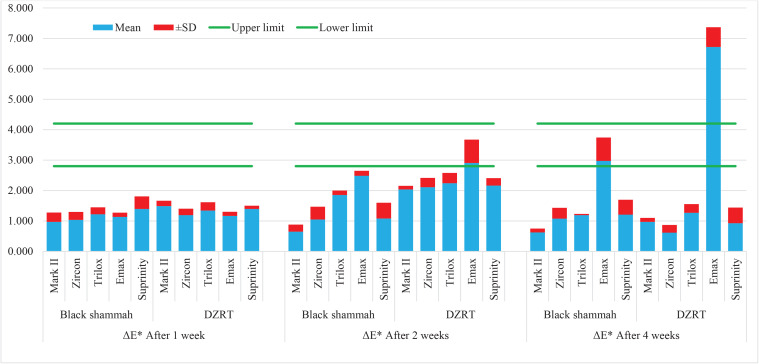
Mean and standard deviation (SD) of color change (ΔE**) values for five CAD/CAM ceramic types after 1, 2, and 4 weeks of exposure to black shammah and DZRT snuff in an in vitro staining model

Figure 1 shows that with the exception of the E. max ceramic, all ceramic types exhibited ΔE** values that were below or marginally higher than the acceptable values. The IPS e.max CAD ceramic, however, demonstrated ΔE** values within the clinically acceptable range (2.8–4.2) following two weeks of exposure to DZRT snuff and black shammah. Furthermore, after four weeks of immersion in DZRT snuff, the ΔE** of the IPS e.max CAD ceramic exceeded the upper limit of acceptable ΔE** values (4.2).

The Kruskal–Wallis test revealed statistically significant differences between the ΔE** values of different ceramic types after two and four weeks of exposure to black shammah (p<0.001 for both) and after one, two, and four weeks of exposure to DZRT snuff (p=0.009, p=0.017, and p<0.001, respectively). However, no statistically significant difference was observed after exposure to black shammah for one week (p=0.100) (Table 2).

**Table 2:** Comparison of mean color change (ΔE**) values between ceramic types at different time intervals exposure and types of smokeless tobacco

*Time*	*Smokeless tobacco*	*Ceramic type*	*Median (IQR)*	*Kruskal–Wallis H*	*df*	*p ^a^*
**After one week**	Black shammah	VITABLOCS Mark II	0.912 (0.606–1.325)	7.788	Δ	0.100
		Zirconia	1.106 (0.698–1.324)			
		VITA TriLuxe	1.176 (0.970–1522)			
		IPS e.max CAD	1.080 (1.042–1.185)			
		VITA Suprinity	1.420 (0.995–1.922)			
	DZRT snuff	VITABLOCS Mark II	1.442 (1.398 –1.542)	13.560	4	0.009
		Zirconia	1.120 (1.068 – 1.259)			
		VITA TriLuxe	1.507 (1.070–1.589)			
		IPS e.max CAD	1.130 (1.025–1.350)			
		VITA Suprinity	1.354 (1.281–1.507)			
**After two weeks**	Black shammah	VITABLOCS Mark II	0.783 (0.324–0.846)	39.262	4	<0.001
		Zirconia	0.802 (0.738–1.653)			
		VITA TriLuxe	1.817 (1.711–1.924)			
		IPS e.max CAD	2.502 (2.295–2.560)			
		VITA Suprinity	0.906 (0.782–1.782)			
	DZRT snuff	VITABLOCS Mark II	2.055 (1.879–2.129)	12.037	4	0.017
		Zirconia	2.097 (1.745–2.487)			
		VITA TriLuxe	2.464 (1.853–2.537)			
		IPS e.max CAD	2.763 (2.169–3.829)			
		VITA Suprinity	2.118 (1.941–2.454)			
**After four weeks**	Black shammah	VITABLOCS Mark II	0.696 (0.442–0.718)	34.748	4	<0.001
		Zirconia	0.957 (0.696–1.544)			
		VITA TriLuxe	1.175 (1.162–1.247)			
		IPS e.max CAD	2.928 (2.183–3.266)			
		VITA Suprinity	1.097 (0.930–1.851)			
	DZRT snuff	VITABLOCS Mark II	0.918 (0.872–1.151)	33.929	4	<0.001
		Zirconia	0.638 (0.308–0.807)			
		VITA TriLuxe	1.197 (0.997–1.662)			
		IPS e.max CAD	6.718 (6.296–7.514)			
		VITA Suprinity	0.628 (0.464–1.381)			

IQR: interquartile range.

Pairwise comparisons after one week of exposure to black shammah were not performed because the overall Kruskal–Wallis test was non-significant. After one week of immersion in DZRT snuff, the ΔE^**^ value of VITABLOCS Mark II was significantly different from those of zirconia (p=0.011) and IPS e.max CAD (p=0.003). Additionally, a significant difference was found between zirconia and VITA Suprinity (p=0.048), between VITA TriLuxe and IPS e.max CAD (p=0.038), and between IPS e.max CAD and VITA Suprinity (p=0.015) (Table 3).

**Table 3:** Pairwise comparisons of mean color change (ΔE**) between ceramic types after exposure to different time exposure and different types of smokeless tobacco

*Time*	*Ceramic type*	*Smokeless tobacco*			
		*Black shammah*		*DZRT snuff*	
		*Median* *difference*	*p ^a^*	*Median* *difference*	*p ^a^*
**After one week**	VITABLOCS Mark II vs Zirconia	-0.193	NA	0.322	0.011
	VITABLOCS Mark II vs VITA TriLuxe	-0.264	NA	-0.065	0.349
	VITABLOCS Mark II vs IPS e.max CAD	-0.167	NA	0.313	0.003
	VITABLOCS Mark II vs VITA Suprinity	-0.507	NA	0.088	0.570
	Zirconia vs VITA TriLuxe	-0.071	NA	-0.387	0.107
	Zirconia vs IPS e.max CAD	0.026	NA	-0.009	0.645
	Zirconia vs VITA Suprinity	-0.314	NA	-0.233	0.048
	VITA TriLuxe vs IPS e.max CAD	0.096	NA	0.378	0.038
	VITA TriLuxe vs VITA Suprinity	-0.243	NA	0.154	0.712
	IPS e.max CAD vs VITA Suprinity	-0.340	NA	-0.224	0.015
**After two weeks**	VITABLOCS Mark II vs Zirconia	-0.019	0.269	-0.042	0.634
	VITABLOCS Mark II vs VITA TriLuxe	-1.034	<0.001	-0.409	0.167
	VITABLOCS Mark II vs IPS e.max CAD	-1.720	<0.001	-0.708	0.001
	VITABLOCS Mark II vs VITA Suprinity	-0.123	0.080	-0.063	0.381
	Zirconia vs VITA TriLuxe	-1.015	0.006	-0.367	0.365
	Zirconia vs IPS e.max CAD	-1.701	<0.001	-0.665	0.007
	Zirconia vs VITA Suprinity	-0.104	0.519	-0.021	0.690
	VITA TriLuxe vs IPS e.max CAD	-0.686	0.094	-0.298	0.072
	VITA TriLuxe vs VITA Suprinity	0.911	0.037	0.346	0.612
	IPS e.max CAD vs VITA Suprinity	1.597	<0.001	0.644	0.021
**After four weeks**	VITABLOCS Mark II vs Zirconia	-0.260	0.044	0.280	0.158
	VITABLOCS Mark II vs VITA TriLuxe	-0.479	0.001	-0.279	0.001
	VITABLOCS Mark II vs IPS e.max CAD	-2.232	<0.001	-5.800	<0.001
	VITABLOCS Mark II vs VITA Suprinity	-0.400	0.022	0.289	0.075
	Zirconia vs VITA TriLuxe	-0.219	0.222	-0.559	0.075
	Zirconia vs IPS e.max CAD	-1.972	<0.001	-6.080	<0.001
	Zirconia vs VITA Suprinity	-0.140	0.788	0.009	0.712
	VITA TriLuxe vs IPS e.max CAD	-1.753	0.013	-5.521	0.025
	VITA TriLuxe vs VITA Suprinity	0.079	0.340	0.568	0.158
	IPS e.max CAD vs VITA Suprinity	1.832	0.001	6.089	<0.001

a Pairwise comparisons between every two ceramic types using ***post hoc*** test with correction. NA: not calculated.

After two weeks of exposure to black shammah, the ΔE^**^ of VITABLOCS Mark II was significantly different from those of VITA TriLuxe and IPS e.max CAD (p<0.001 for both). Similarly, the ΔE^**^ of zirconia was significantly different from those of VITA TriLuxe Forte (p=0.006) and IPS e.max CAD (p<0.001). The ΔE^**^ of VITA Suprinity was significantly different from those of VITA TriLuxe (p=0.037) and IPS e.max CAD (p<0.001). After two weeks of immersion in DZRT snuff, only the ΔE^**^ of VITABLOCS Mark II was significantly different from that of IPS e.max CAD (p<0.001), zirconia from that of IPS e.max CAD (p=0.007), and IPS e.max CAD from that of VITA Suprinity (p=0.021) (Table 3).

Almost all pairwise comparisons revealed statistically significant differences after four weeks of exposure to black shammah except between Zirconia versuss VITA TriLuxe, Zirconia versus VITA Suprinity, and Zirconia versus VITA Suprinity with p values of 0.222, 0.788, and 0.340, respectively. After four weeks of immersion in DZRT snuff, a statically significant differences were observed between VITABLOCS Mark II and VITA TriLuxe (p<0.001) and IPS e.max CAD (p<0.001). Moreover, Zirconia exhibited a significant difference from IPS e.max CAD (p<0.025). IPS e.max CAD showed a significant difference from VITA Suprinity (p<0.001) (Table 3).

Statistically significant differences in the ΔE** values of VITABLOCS Mark II ceramic were observed between black shammah and DZRT snuff types after one week (p=0.001), two weeks (p<0.001), and four weeks (p<0.001) of exposure. The ΔE** values of Zirconia ceramic exposed to black shammah and DZRT snuff showed statistically significant differences after two and four weeks of exposure (p<0.001 and 0.008, respectively). The ΔE** values of VITA TriLuxe and VITA Suprinity ceramics exhibited a statistically significant difference only after two weeks of exposure (p=0.010 and <0.001, respectively). In the case of IPS e.max CAD ceramic, a statistically significant difference was observed only after four weeks of exposure (p<0.001) (Table 4).

**Table 4:** Comparison of mean color change (ΔE**) values between smokeless tobacco types for each ceramic material after different exposures intervals

*Ceramic type*	*Smokeless tobacco*	*After one week*		*After two weeks*		*After four weeks*	
		*Median (IQR)*	*p ^a^*	*Median (IQR)*	*p ^a^*	*Median (IQR)*	*p ^a^*
**VITABLOCS Mark II**	Black shammah	0.912 (0.606–1.325)	0.001	0.783 (0.324–0.846)	<0.001	0.696 (0.442–0.718)	<0.001
	DZRT snuff	1.442 (1.398–1.542)		2.055 (1.879–2.129)		0.918 (0.872–1.151)	
**Zirconia**	Black shammah	1.106 (0.698–1.324)	0.402	0.802 (0.738–1.653)	<0.001	0.957 (0.696–1.544)	0.008
	DZRT snuff	1.120 (1.068–1.259)		2.097 (1.745–2.487)		0.638 (0.308–0.807)	
**VITA TriLuxe**	Black shammah	1.176 (0.970–1522)	0.446	1.817 (1.711–1.924)	0.010	1.175 (1.162–1.247)	0.939
	DZRT snuff	1.507 (1.070–1.589)		2.464 (1.853–2.537)		1.197 (0.997–1.662)	
**IPS e.max CAD**	Black shammah	1.080 (1.042–1.185)	1.000	2.502 (2.295–2.560)	1.000	2.928 (2.183–3.266)	<0.001
	DZRT snuff	1.130 (1.025–1.350)		2.763 (2.169–3.829)		6.718 (6.296–7.514)	
**VITA Suprinity**	Black shammah	1.420 (0.995–1.922)	0.879	0.906 (0.782–1.782)	<0.001	1.097 (0.930–1.851)	0.223
	DZRT snuff	1.354 (1.281–1.507)		2.118 (1.941–2.454)		0.628 (0.464–1.381)	

a Mann–Whitney U.

The Kruskal–Wallis H test indicated significant differences in the ΔE^**^ values of VITABLOCS Mark II (p=0.030), VITA TriLuxe (p<0.001), and IPS e.max CAD (p<0001) between time points after exposure to black shammah. For all ceramic types, significant differences in ΔE^**^ between time points were found after immersion in DZRT snuff (all p<0.001) for VITABLOCS Mark II, Zirconia, VITA TriLuxe, IPS e.max CAD and VITA Suprinity (Table 5).

**Table 5:** Comparison of mean color change (ΔE**) values across different time points for each ceramic type after exposure to different types of smokeless tobacco

*Ceramic type*	*Time*	*Smokeless tobacco*			
		*Black shammah*		*DZRT snuff*	
		*Median (IQR)*	*p ^a^*	*Median (IQR)*	*p ^a^*
**VITABLOCS Mark II**	After one week	0.912 (0.606–1.325)	0.030	1.442 (1.398–1.542)	<0.001
	After two weeks	0.783 (0.324–0.846)		2.055 (1.879–2.129)	
	After four weeks	0.696 (0.442–0.718)		0.918 (0.872–1.151)	
**Zirconia**	After one week	1.106 (0.698–1.324)	0.976	1.120 (1.068–1.259)	<0.001
	After two weeks	0.802 (0.738–1.653)		2.097 (1.745–2.487)	
	After four weeks	0.957 (0.696–1.544)		0.638 (0.308–0.807)	
**VITA TriLuxe**	After one week	1.176 (0.970–1522)	<0.001	1.507 (1.070–1.589)	<0.001
	After two weeks	1.817 (1.711–1.924)		2.464 (1.853–2.537)	
	After four weeks	1.175 (1.162–1.247)		1.197 (0.997–1.662)	
**IPS e.max CAD**	After one week	1.080 (1.042–1.185)	<0.001	1.130 (1.025–1.350)	<0.001
	After two weeks	2.502 (2.295–2.560)		2.763 (2.169–3.829)	
	After four weeks	2.928 (2.183–3.266)		6.718 (6.296–7.514)	
**VITA Suprinity**	After one week	1.420 (0.995–1.922)	0.082	1.354 (1.281–1.507)	<0.001
	After two weeks	0.906 (0.782–1.782)		2.118 (1.941–2.454)	
	After four weeks	1.097 (0.930–1.851)		0.628 (0.464–1.381)	

a Kruskal–Wallis test.

Pairwise comparisons between different times of exposure to black shammah revealed that the ΔE^**^ of VITABLOCS Mark II and IPS e.max CAD did not differ significantly between two and four weeks of exposure (p=0.507 and p=0.306, respectively), but for the other time intervals for these materials, and for VITA TriLuxe across all intervals, differences were significant (all p<0.050). For zirconia and VITA Suprinity, pairwise comparisons between time intervals were not performed because the overall Kruskal–Wallis test was non-significant (p=0.976 and p=0.082, respectively). After immersion in DZRT snuff, significant differences in ΔE^**^ were found between every two time points for all ceramic types (all p<0.05), except for VITA TriLuxe between one and four weeks (p=0.959) and VITA Suprinity between one and four weeks (p=0.126) (Supplementary file Table 2).

### Surface roughness measurements

R_a_ values were 1.61, 1.98, 2.49, 2.89, and 2.92 in black shammah (Supplementary file Figure 2) and 1.65, 1.39, 1.89, 2.92, and 1.78 in DZRT snuff (Supplementary file Figure 3).

### VITA classic shades assessments

Supplementary file Table 3 indicates that significant changes in VITA classic shades were observed after two and four weeks of exposure to black shammah, with p=0.048 and 0.008, respectively, and after one, two, and four weeks of immersion in DZRT snuff, with p=0.001, 0.010, and 0.001, respectively. A significant difference was found at all different periods (p<0.05) but not at one week after exposure to black shammah (p=0.195) (Supplementary file Table 3).

## DISCUSSION

Critical properties for the aesthetic longevity of new dental ceramic materials include good color stability, which is defined as a minimally perceptible color change over time in the oral environment along with low R_a_ values given that a smooth surface texture is essential for minimizing stain adsorption and maintaining appearance^27^. Previous findings have indicated that CAD/CAM ceramics exhibit clinically unacceptable ΔE^**^ and increased R_a_ values when exposed to ST products^18,23^. In this study, we investigated the effects of black shammah and DZRT snuff on the color stability and surface profilometry results of five CAD/CAM ceramics over one, two, and four weeks of exposure.

This study results demonstrated that the ΔE^**^ of most samples remained within clinically acceptable thresholds (ΔE^**^ <4.2). Among the materials evaluated in the present study, IPS e.max CAD showed the most pronounced discoloration, with a ΔE^**^ of 6.718 after four weeks of exposure to DZRT snuff. By contrast, the smallest color variations were shown by VITABLOCS Mark II (ΔE^**^ =0.623) when stained with black shammah and by zirconia (ΔE^**^ =0.614) when exposed to DZRT snuff for the same period. Our findings align with those of Al Moaleem et al.^17^ who found that black and white ST affected the color stability of various CAD/CAM ceramic materials, with Ceramill Zolid PS zirconia demonstrating the lowest color change. However, Daghrery et al.^18^ reported a low ΔE^**^ (3.9) for lithium disilicate glass ceramics after two weeks of exposure to ST snuff. This variability may be attributed to differences in the shape of the assessed samples, use of brushing and thermocycling during staining, and the type of tobacco staining agents.

The findings of this study led to our rejection of the first part of the null hypothesis, which proposed that color changes do not significantly differ among various ceramic types after exposure to the STs black shammah and DZRT snuff. As shown in Table 3, notable variations were observed between ceramic types when exposed to different ST products. Additionally, significant differences in discoloration effects between the two ST types across ceramic specimens were observed, as illustrated in Table 4. These changes may be attributed to the variation in the deposition ability of the inorganic compounds, including heavy metals, such as lead, cadmium, and arsenic, as well as other particulate matter, present in various ST products^23,28^. Over time, these substances can accumulate on ceramic surfaces, leading to visible discoloration^29^.

Among the tested materials, leucite-reinforced feldspathic glass (VITABLOCS^®^ Mark II) exhibited the least color change after exposure to black shammah and DZRT snuff. This finding aligns with the results of prior research by Alqahtani et al.^29^ and Moaleem et al.^30^ (2021), who reported that feldspathic ceramics demonstrate good color stability because of their lack of glass fillers, which helps maintain surface smoothness post immersion. On the basis of these results, we recommend prioritizing feldspathic ceramics for patients using ST products to minimize aesthetic compromises.

Our findings revealed that the ΔE^**^ values of all CAD/CAM materials increased significantly with the prolongation of staining times. Statistically significant differences were observed for VITABLOCS Mark II, VITA TriLuxe, and IPS e.max CAD after exposure to black shammah, as well as for all ceramics immersed in DZRT snuff. Consequently, we rejected the second part of the null hypothesis, as significant variations in ΔE^**^ were evident across the one-, two-, and four-week intervals. Our results align with those of Colombo et al.^31^ who found that in zirconia, acidic drinks initially caused minimal discoloration (ΔE^**^ <3.3) but subsequent coffee exposure affected color stability, and with those of Moaleem et al.^30^ who noted that prolonged staining durations significantly affected the optical properties of VITABLOCS Mark II and Ceramill Zolid PS.

Surface roughness remarkably influences dental ceramics by degrading optical properties and mechanical stability. It reduces translucency and increases crack propagation. Elevated roughness (R_a_ >0.2 μm) accelerates aging, weakens biomechanical integrity, and impairs aesthetics, leading to unacceptable color changes (ΔE^**^ >3.0)^32^. Rough surfaces also increase plaque accumulation by 70% and wear on opposing teeth by 40%, as well as act as stress concentrators, leading to restoration failure through microcracks^14^.

Measurements of R_a_ showed notable differences among ceramic materials exposed to black shammah and DZRT snuff, as depicted in Supplementary file Figures 2 and 3. Among the materials, VITABLOCS Mark II had the highest resistance, with R_a_ values of 1.61 μm when exposed to black shammah and 1.65 μm when immersed in DZRT snuff. Among materials, IPS e.max CAD was the most affected, showing high roughness (2.89 and 2.92 μm). VITA Suprinity displayed moderate susceptibility, with R_a_ values of 2.92 and 1.78 μm under exposure to black shammah and DZRT, respectively. These results align with those of Moaleem et al.^33^ who reported an R_a_ value of 1.26 μm for VITABLOCS after khat immersion and thermocycling. However, they exceed the values reported by Aldosari et al.^19^ (0.56 μm for VITABLOCS and 0.66 μm for VITA Suprinity), Egilmez et al.^34^ (0.012 μm for hybrid resin), and Vasiliu et al.^35^ (0.12 μm for CAD/CAM materials). Variations are attributed to differences in experimental conditions, staining agents, exposure durations, thermocycling protocols, measurement techniques, and material composition.

The VITA classical color shade guide is a standardized system used in dentistry for selecting tooth shades for aesthetic restorations, such as crowns and veneers. It includes 16 shade tabs organized into four hue groups: A (reddish-brown), B (reddish-yellow), C (grayish), and D (reddish-gray)^21^. Each group has numerical suffixes indicating chroma; for instance, in group A, shades range from the lightest A1 to the darkest A4^22^. In this study, we rejected the third part of the null hypothesis stating that staining agents do not affect the shades of specimens. Significant, time-dependent darkening was noted, although patterns varied by ST product. Compositional variations likely explain these differences: the high pH/additive contents of black shammah may accelerate oxidative darkening, whereas the fine particulates of DZRT could enable deep staining followed by leaching^18^.

In accordance with the VITA classical shade guide, shade A2 was the most prevalent at baseline for both ST types (62.0% for black shammah and 58.0% for DZRT snuff). Immersion induced remarkable, time-dependent darkening, though its patterns differed between products. For materials immersed in black shammah, the prevalence of shade A2 decreased from 62.0% at baseline to 42.0% by week 4 (p=0.008), whereas those of shades A3 and B2 increased. By contrast, DZRT snuff caused an abrupt initial shift, with the prevalence of A2 declining to 24.0% after one week (p=0.001) and that of B1 rising sharply to 76.0%. However, by week 4, the prevalence of B1 decreased to 22.0%, whereas that of A2 returned to 50.0%; another study reported similar changes in similar materials after two weeks of ST staining^29^.

The observed time-dependent darkening patterns induced by tobacco immersion that we observed in this work align with those found in studies on material discoloration. The remarkable initial shift away from shade A2 seen in DZRT snuff, followed by a partial reversal by week 4, was similar to the findings showing that zirconia darkened after 24 h in chlorhexidine but then lightened at 72 h and seven days of immersion, suggesting potential reversible adsorption or leaching^36^. Similarly, the partial reversal of A2 in DZRT snuff at week 4 (50.0%) align with findings indicating that the shade of only 50% of zirconia samples remained at A2 after two weeks^30^. While some studies noted a shift to A2 in zirconia, the significant reduction in A2 prevalence observed here, especially the sharp decline to a prevalence of 24.0% after one week of immersion in DZRT, resulted in clinically relevant changes^37^. These findings are consistent with those of research that found color differences exceeding perceptibility thresholds using the Vita classic shade guide^30^. The use of this guide supports the reliability of the detected shade changes given that its shade matching accuracy does not significantly differ from those of newer guides as a result of its optimized color space distribution^38^.

### Strengths and limitations

This study has several notable strengths. It provides a comprehensive comparative analysis of five widely used CAD/CAM ceramics, evaluating their performance against two distinct and regionally relevant smokeless tobacco products. The experimental design incorporated multiple clinically relevant assessments – quantitative colorimetry (ΔE^**^), surface profilometry (R_a_), and qualitative shade guide matching – alongside simulated aging through thermocycling and brushing. This multi-faceted approach enhances the clinical applicability of the findings.

The present study has several limitations that should be considered when interpreting its findings. As an *in vitro* experiment, it cannot replicate the full complexity of the oral environment, which includes saliva, enzymes, or chewing forces. The four-week exposure and thermocycling treatment that it employed may not reflect long-term clinical effects or other aging factors, like biofilm buildup. Its small, homogeneous sample size limits generalizability, and only two ST varieties, but not other regional or commercial products that might yield different outcomes, were included. This study did not explore how different surface treatments (e.g. polishing vs glazing) might affect results, nor did it assess mechanical properties beyond roughness. Shade matching relied on a single operator, risking bias. These limitations highlight the need for broad clinical studies with extended follow-ups, diverse samples, and expanded outcome measures.

## CONCLUSIONS

The following conclusions were drawn in the present *in vitro* study: R_a_ and ΔE^**^ are directly correlated. As R_a_ (μm) increased, the ΔE^**^ values of the assessed ceramics increased.

The ST products black shammah and DZRT snuff induced measurable color changes (ΔE^**^) in all tested CAD/CAM ceramics, with the most pronounced discoloration observed in lithium disilicate glass ceramic (IPS e.max CAD), exceeding clinically acceptable thresholds after prolonged exposure. By contrast, multilayer zirconia (Ceramill Zolid PS) and feldspathic ceramic (VITABLOCS Mark II) demonstrated superior color stability to other materials, with their ΔE^**^ values remaining below critical. The R_a_ values of all materials increased after exposure to black shammah and DZRT snuff, with the highest values recorded for IPS e.max CAD and the lowest for VITABLOCS Mark II. Clinicians should prioritize zirconia or feldspathic ceramics for patients with ST use to optimize longevity and aesthetics.

## Supplementary Material



## Data Availability

The datasets used and/or analyzed during the current study are available from the author on reasonable request.
