# Supplementary material for: Correlation between the color stability and contact profilometry results of different CAD/CAM ceramic materials after staining with black shammah and DZRT smokeless tobacco
Source: Tob Induc Dis. 2026 Apr 1;24:10.18332/tid/215947. doi: 10.18332/tid/215947 (PMC13044779; doi:10.18332/tid/215947)
Supplement: Supplementary file 1 [file TID-24-50-s1.pdf]

Supplementary Table 1: Materials and devices used in the study

**Material/ Device type**	**Brand name**	**Composition**	**Manufacturers**	**Color/ application per day**
DZRT snuff	DZRT® nicotine pouch– e.g. Highland Berries 6 mg/7.42	Cellulose plant fibers, water, pharma-grade nicotine 3/6/10 mg $pouch^{-1}$, propylene glycol (humectant), sodium carbonate + bicarbonate (pH adjusters), acesulfame K or xylitol (sweeteners), food-grade flavorings, trace NaCl	Badael International Co., Riyadh, Saudi Arabia	White pouch/2 (placed on specimens twice daily, 30 min each)
Black shammah	Black shammah/ 9.37	Mainly manufactured by powdering tobacco along with ash, flavors, oils, calcium oxide, and black pepper	Procured in plastic packages from markets	Black/2
Feldspathic porcelain CAD/CAM blocks	VITABLOCS Mark II	Fine-particle feldspar glass ceramic, low-to-moderate < 50% % leucite content	VITA Zahnfabrik, Bad Säckingen, Germany	A2
Zirconia CAD/CAM	Ceramill Zolid multilayer PS	$ZrO_2 + HFO_2 + Y_2O_3$: ≥99.0, Y_2O_3: 8.5–9.5, HFO_2: ≤5, Al_2O_3: ≤0.5, other oxides: ≤1	AmannGirrbach, Germany	A2
Feldspathic ceramic	VITA Triluxe Forte	SiO_2: 56–64, Al_2O_3: 20–23, Na_2O: 6–9, K_2O: 6–8, CaO: 0.3–0.6, TiO_2: ≤0.1, other oxides: ≤11	VITA Zahnfabrik H. Rauter GmbH & Co. KG, Germany	A2
Lithium disilicate glass	IPS E. max CAD,	SiO_2, Li_2O, K_2O, P_2O_5, ZrO_2, ZnO, Al_2O_3, MgO	Ivoclar Vivadent,	A2

ceramic			Schaan, Liechtenstein.GC,	
zirconia-reinforced lithium silicate ceramic	VITA Suprinity	SiO_2 (56%–64%), Li_2O (15%–21%), ZrO_2 (8%–12%), La_2O_3 (0.1%), and pigments	VITA Zahnfabrik, Bad Säckingen, Germany	A2
Artificial saliva	Unstimulated whole human saliva (pooled, filtered)	≈99 % water; electrolytes Na^+ ~80 mmol L^{-1}, K^+ ~8 mmol L^{-1}, Cl^- ~40 mmol L^{-1}, HCO_3^- 15–25 mmol L^{-1}, Ca^{2+} ~1–2 mmol L^{-1}; proteins ≈ 0.5 g L^{-1} (amylase, mucins, proline-rich proteins, IgA, statherin); pH 6.2–7.6	Collected fresh from healthy volunteers	Clear, slightly opalescent/medium renewed every 24 h
Spectrophotometer	VITA Easyshade® V	Device used to measure wavelength transmitted from one object at a time without being affected by subjective color interferences	VITA Zahnfabrik H. Rauter GmbH & Co. KG, Bad Sackingen, Germany	Color parameter L*, a*, and b* measurements
Surface Roughness Tester	Profilometer	Device recorded graphically the average height of the profile above and below a center line along the given length of a sample	Perthometer M2, Mahr GmbH, Germany	R_a measurement
Shade guide	VITA classic shade guide	Used for the clinical shade selection of ceramic restorations	VITA Zahnfabrik H. Rauter GmbH & Co. KG, Bad Sackingen, Germany	A1–D4® shade guide

Supplementary Table 2: Pairwise comparisons of color change (ΔE_{**}) between time points for each ceramic type after exposure to different types of smokeless tobacco

		Smokeless tobacco			
**Ceramic type**	**Time**	**Black shammah**		**DZRT snuff**	
		**Median difference**	**p Value [a]**	**Median difference**	**p Value [a]**
VITABLOCS Mark II	After one week vs after two weeks	0.130	0.059	-0.613	0.011
	After one week vs after four weeks	0.216	0.011	0.524	0.011
	After two weeks vs after four weeks	0.086	0.507	1.137	<0.001
Zirconia	After one week vs after two weeks	0.304	NA	-0.977	0.011
	After one week vs after four weeks	0.149	NA	0.483	0.011
	After two weeks vs after four weeks	-0.155	NA	1.460	<0.001
VITA TriLuxe	After one week vs after two weeks	-0.640	<0.001	-0.957	<0.001
	After one week vs after four weeks	0.001	0.019	0.311	0.959
	After two weeks vs after four weeks	0.641	<0.001	1.267	<0.001
IPS e.max CAD	After one week vs after two weeks	-1.423	0.001	-1.633	0.011
	After one week vs after four weeks	-1.848	<0.001	-5.588	<0.001
	After two weeks vs after four weeks	-0.426	0.306	-3.955	0.011
VITA Suprinity	After one week vs after two weeks	0.514	NA	-0.765	0.002
	After one week vs after four weeks	0.323	NA	0.725	0.126
	After two weeks vs after four weeks	-0.191	NA	1.490	<0.001

[a] Pairwise Comparisons between every two different time using Post hoc test with correction. NA-not calculated

Supplementary Table 3: Comparison of VITA classical shade guide assessments at baseline and after 1, 2, and 4 weeks of exposure to black shammah and DZRT snuff

Smokeless tobacco	VITA classic	Baseline	After one week		After two weeks		After four weeks	
		No. (%)	No. (%)	p Value [1]	No. (%)	p Value [2]	No. (%)	p Value [3]
Black shammah	A2	31 (62.0)	27 (54.0)	0.195	24 (48.0)	0.048	21 (42.0)	0.008
	B2	2 (4.0)	2 (4.0)		6 (12.0)		8 (16.0)	
	A3	17 (34.0)	21 (42.0)		20 (40.0)		21 (42.0)	
DZRT snuff	A2	29 (58.0)	31 (24.0)	0.001	25 (30.0)	0.010	23 (50.0)	0.001
	B1	13 (42.0)	15 (76.0)		23 (66.0)		24 (22.0)	
	B2	8 (4.0)	4 (0.0)		2 (4.0)		3 (8.0)	

[1] p Value for comparison between the baseline and after one week

[2] p Value for comparison between the baseline and after two weeks

[3] p Value for comparison between the baseline and after four weeks

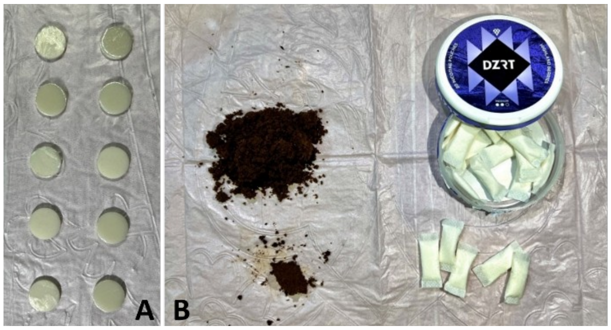

Supplementary Figure 1. Forms of ceramic CAD/CAM materials (A) and black shammah and DZRT snuff (B).

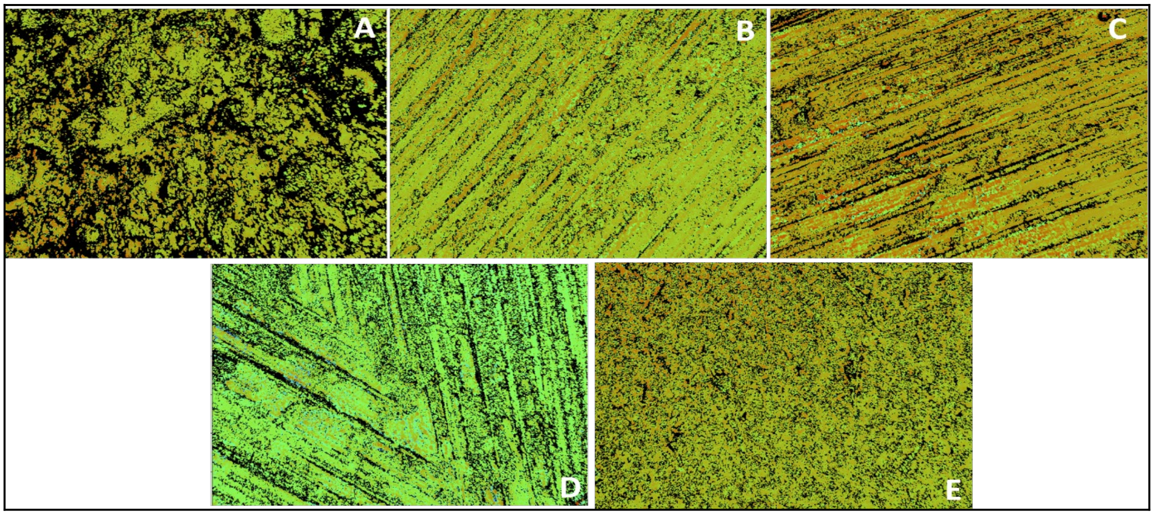

Supplementary Figure 2. Representative surface roughness R_a images of glazed CAD/CAM ceramic specimens after four weeks of staining with black shammah in an *in vitro* study: (A) VITABLOCS Mark II, (B) Ceramill Zolid multilayer PS, (C) VITA Triluxe Forte, (D) IPS e.max .CAD, and (E) VITA Suprinity

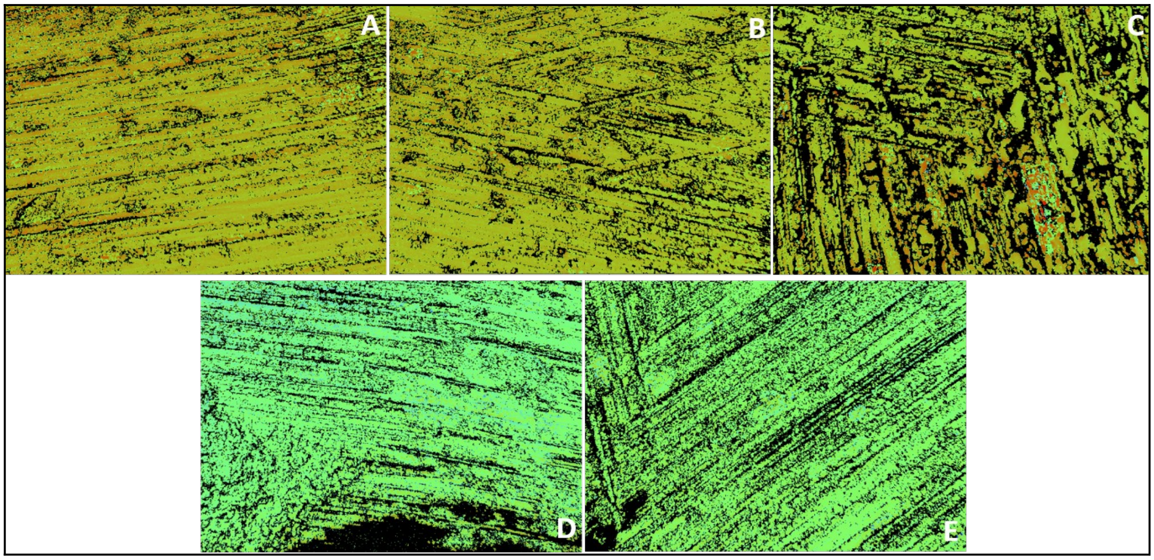

Supplementary Figure 3. Representative surface roughness R_a images of glazed CAD/CAM ceramic specimens after four weeks of staining with DZRT snuff in an *in vitro* study: (A) VITABLOCS Mark II, (B) Ceramill Zolid multilayer PS, (C) VITA Triluxe Forte, (D) IPS e.max CAD, and (E) VITA Suprinity.

©2026 AlWadei M.H.
